# Hydrophobicity Does Not Affect Water Slip: Insights from Slip Length Mapping

**DOI:** 10.1021/acs.nanolett.6c01403

**Published:** 2026-05-15

**Authors:** Haruya Ishida, Koji Takahashi, Vishwanath Ganesan, Nenad Miljkovic, Hideaki Teshima

**Affiliations:** † Department of Aeronautics and Astronautics, 12923 Kyushu University, Nishi-Ku, Motooka 744, Fukuoka 819-0395, Japan; ‡ International Institute for Carbon-Neutral Energy Research (WPI-I2CNER), Kyushu University, Nishi-Ku, Motooka 744, Fukuoka 819-0395, Japan; ¶ Department of Mechanical Science and Engineering, 14589 University of Illinois at Urbana−Champaign, Urbana, Illinois 61801, United States; § Materials Research Laboratory, University of Illinois at Urbana−Champaign, Urbana, Illinois 61801, United States; ∥ Department of Electrical and Computer Engineering, University of Illinois at Urbana−Champaign, Urbana, Illinois 61801, United States; ⊥ Institute for Sustainability, Energy and Environment (iSEE), University of Illinois at Urbana−Champaign, Urbana, Illinois 61801, United States; # Air Conditioning and Refrigeration Center, University of Illinois at Urbana−Champaign, Urbana, Illinois 61801, United States

**Keywords:** slip length, water, AFM, graphite, hydrophobic surface, nanobubble

## Abstract

While slip at solid–water interfaces has attracted broad interest, slip lengths reported in experiments—especially on hydrophobic surfaces (i.e., contact angles >90°)—vary widely due to surface topographical and chemical heterogeneity, hindering quantitative understanding of the true slip length. Using highly sensitive frequency-modulation atomic force microscopy, we achieved simultaneous nanoscale mapping of slip length and surface topography, with a 159-fold improvement in slip length detection sensitivity. True slip lengths measured on flat regions of various substrates were found to be almost zero, following a scaling rule predicted by molecular dynamics simulations. As the only exception, a large slip length of 43.2 ± 5.8 nm was measured on graphite in deionized water, but it vanished upon immersion in electrolyte solutions. This unique behavior was rationalized by graphite’s atomic-scale smoothness, chemical homogeneity, and ion adsorption. Our method experimentally advances a unified picture of fluid slip.

## Graphical abstract

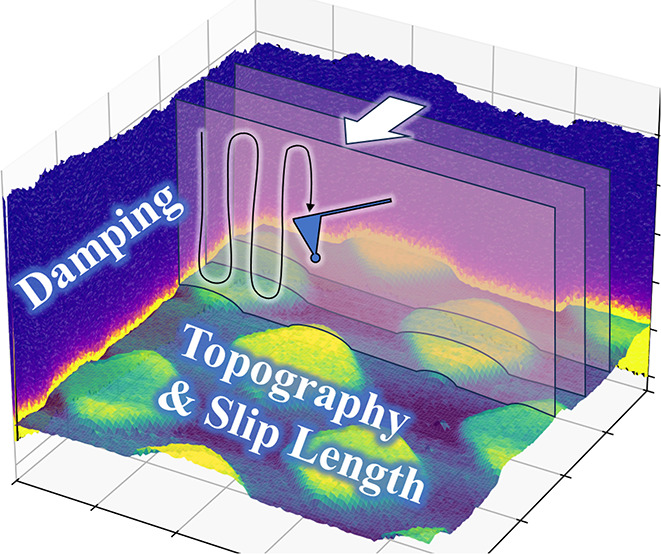

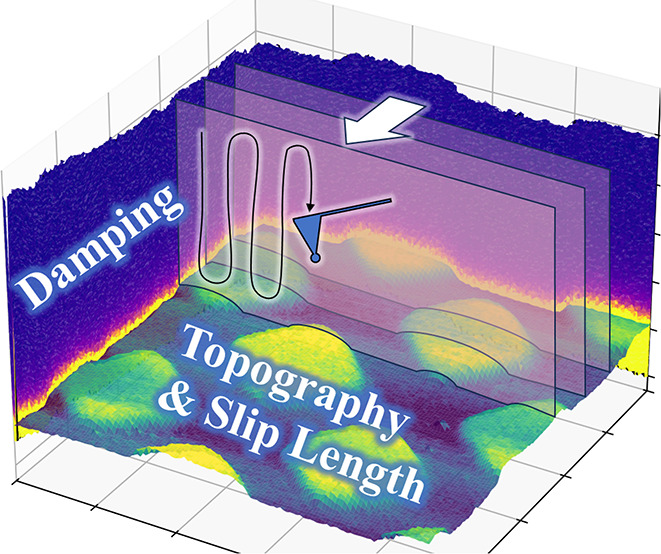

The no-slip boundary condition, which assumes that the fluid velocity at a solid wall is zero, has long been regarded as an implicit premise in fluid mechanics. However, recent experiments have reported the occurrence of boundary slip, where fluids slip along solid surfaces with a finite velocity.^1–7^ Slip is quantified by the slip length *b*, defined in terms of the slip flow velocity *v*_s_ and the shear rate ∂*v*/∂*z*, as follows:^8^

1 $$v_{s}=b{\left(\frac{\partial v}{\partial z}\right)}_{z=0}$$

Solid–liquid friction is drastically reduced when slip occurs. For instance, the flow rate of water through carbon nanotubes^9^ and hydrophobic fluorous nanochannels^10^ has been reported to exceed continuum predictions by orders of magnitude. Such enhancements are decisive factors for the performance of nanofluidic devices, in which interfacial effects are dominant. Thus, efficient molecular transport realized by the control of slip is highly desired for breakthroughs in desalination,^10^ lab-on-a-chip technologies,^11^ osmotic power generators,^12,13^ and on-chip cooling.^14,15^

Previous studies have suggested that solid–liquid interfacial properties such as wettability,^16^ surface roughness,^17^ surface charge,^18^ surface free energy distribution,^19^ and surface nanobubbles^20^ strongly influence the slip length. However, despite extensive efforts, their quantitative relationships remain unclear. In particular, unlike hydrophilic surfaces (i.e., contact angles <90°), for which the slip length has generally been reported to be close to zero in both experimental and simulation studies, there is no clear consensus on slip lengths for hydrophobic surfaces (i.e., contact angles >90°). A scaling law relating the slip length *b* to the intrinsic contact angle *θ* is derived as follows:^16^

2 $$b=\frac{C}{{\left(1+\cos\theta \right)}^{2}}$$

where *C* is a prefactor with dimensions of length. Slip lengths obtained from molecular dynamics (MD) simulations are well fit by eq 2 with *C* = 0.41 nm, indicating almost no slip even at intrinsic contact angles of 120°. A contact angle of approximately 120° is achieved on a surface where −CF_3_ groups, which have the lowest surface free energy among surface-terminating groups, are arranged in a hexagonal close-packed structure, and is known as the near-maximum water contact angle achievable on a chemically homogeneous flat surface.^21^ In contrast, when experimental slip length data from various studies are compiled, they show significant deviation from the MD-predicted scaling law with *C* = 0.41 nm. For example, Wu et al. compiled 11 experimental studies and reported *C* = 6.0 nm, corresponding to slip lengths of tens of nanometers above 90°.^22^ We additionally incorporated 14 experimental studies into Wu et al.’s plot (Figure 1). On hydrophilic surfaces, the data agreed well with the curve for *C* = 0.41 nm, as discussed above. In contrast, the data obtained on hydrophobic surfaces deviated from the *C* = 0.41 nm curve and even from the *C* = 6.0 nm curve. Moreover, although not included in Figure 1, a much larger value (∼1 μm) has been measured.^23^

**Figure 1:**
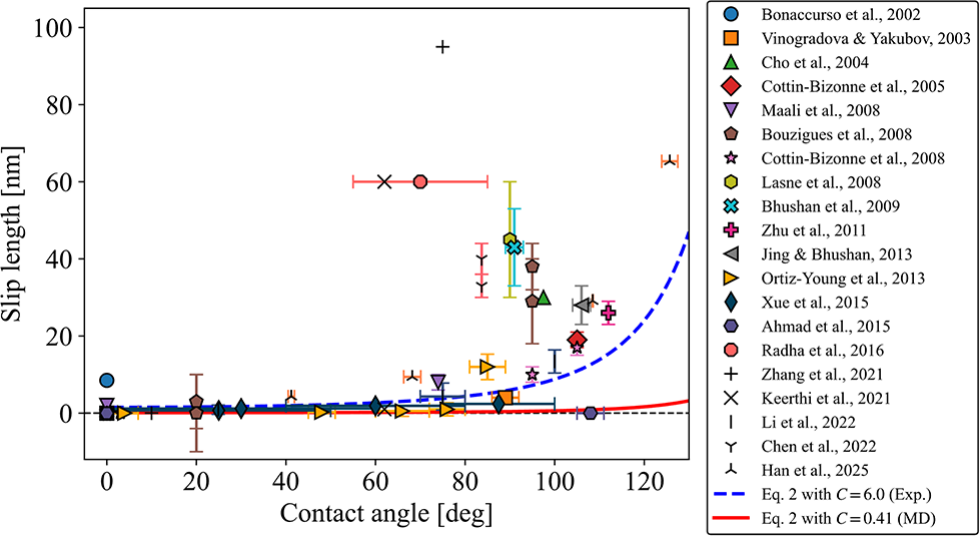
Experimental slip length data of solid–water interfaces from previous studies^2,6,7^ ^,^ ^24–40^ plotted as a function of contact angle. The red dashed line represents the theoretical curve given by eq 2 with *C* = 0.41 nm and the blue dashed line corresponds to *C* = 6.0 nm. The slip length values are summarized in Table S1 of the Supporting Information. Adapted with permission from ref 22. Copyright 2016 Society of Petroleum Engineers.

This discrepancy between experiments and MD analyses is likely due to a complex interplay of multiple factors, not only measurement artifacts^41^ but also nanoscale interfacial effects, such as reduced friction caused by nanobubbles^20^ and apparent slip arising from nanoscopic surface roughness.^42^ No existing technique can measure slip lengths while explicitly accounting for these interfacial effects. As a result, the measured (apparent) slip lengths can deviate from the intrinsic values, making experimental investigations of true slip highly challenging. In other words, we still cannot answer the simple yet long-standing question: “Does water slip on hydrophobic surfaces?”

To date, atomic force microscopy (AFM) provides the highest lateral spatial resolution for slip length measurements.^4–7^ However, in practice, the lateral resolution is typically limited to approximately 10 μm because of limited detection sensitivity, which is still insufficient for accurate slip length measurements.

In this study, we dramatically improve the spatial resolution of slip length measurements by employing highly sensitive frequency-modulation (FM) AFM. In AFM-based slip measurements, the viscous drag force *F*_h_ acting on the sphere of an AFM probe tip near a planar substrate is given by^5^

$$F_{h}=-\gamma_{\mathrm{tip}}ḣ=-\frac{6\pi \eta R^{2}ḣ}{h}f^{*}\left(h,b_{t},b_{s}\right)$$ 3

where *γ*_tip_ is the viscous damping coefficient acting on the probe tip, *h* is the probe-substrate distance, *ḣ* is the probe-substrate approach velocity, *η* is the viscosity of the surrounding medium, *R* is the radius of curvature of the probe tip, *b*_t_ and *b*_s_ are the slip lengths at the probe tip and substrate surfaces, respectively, and f* is a correction factor representing the reduction of viscous drag due to slip. eq 3 is the lubrication-theory expression for the hydrodynamic force between a sphere and a plane with slip boundary conditions. Experimentally, *h*(*t*) is obtained from the measured probe-substrate distance trajectory, and *ḣ* is calculated numerically from this *h*(*t*) data. The correction factor f*, obtained by integrating the lubrication pressure over the sphere-plane gap, is given by^5^

$$f^{*}\left(h,b_{t},b_{s}\right)=-\frac{2Ah}{BC}-\frac{2h}{C-B}\left[\frac{\left(B+h\right)\left(B-A\right)}{B^{2}}\ln\left(1+\frac{B}{h}\right)-\frac{\left(C+h\right)\left(C-A\right)}{C^{2}}\ln\left(1+\frac{C}{h}\right)\right]$$ 4

where the auxiliary quantities *A*, *B*, and *C* are defined as

$$A=b_{t}+b_{s},B=2\left(b_{t}+b_{s}+\sqrt{b_{t}^{2}-b_{t}b_{s}+b_{s}^{2}}\right),,C=2\left(b_{t}+b_{s}-\sqrt{b_{t}^{2}-b_{t}b_{s}+b_{s}^{2}}\right)$$ 5

In conventional contact-mode AFM, *F*_h_ is determined from the static deflection of the cantilever and the viscous drag scales with *R* ^2^ as shown in eq 3. Consequently, when the probe radius is reduced, the detection sensitivity rapidly decreases. Although the smallest probe radius reported to date is *R* = 1.9 μm,^6^ probe tips with radii of approximately 10 μm^7^ are typically used, and further miniaturization remains challenging.

To overcome this limitation, we employ FM-AFM,^43^ in which the cantilever is continuously oscillated at its resonance frequency. In this mode, the energy dissipated by viscous drag is quantified as the damping coefficient *γ*. Because the resonance amplifies the viscous drag, the sensitivity to it is significantly enhanced, thereby compensating for the signal-to-noise degradation proportional to *R* ^2^. Then, *γ*_tip_ can be obtained from the oscillation amplitudes *A*_bulk_ and *A*(*h*) (see Supporting Information I for details):

6 $$\gamma_{\mathrm{tip}}\left(h,b_{t},b_{s}\right)=\gamma_{\mathrm{bulk}}\left(\frac{A_{\mathrm{bulk}}}{A\left(h\right)}-1\right)$$

where the subscript ”bulk” denotes the value at sufficiently large *h*. This relation follows from *γ*_total_ = *γ*_tip_ + *γ*_bulk_, *Q* = *k*/(*ωγ*_total_), and the proportionality *A* ∝ *Q* for constant drive power, as derived in Supporting Information I. In conventional FM-AFM, the oscillation amplitude is kept constant (*A*(*h*) = *A*_bulk_ = const.) by an automatic gain control (AGC) circuit. However, in our method, the AGC is turned off and instead the drive power is kept constant, allowing the amplitude reduction *A*(*h*) to directly reflect the magnitude of the damping coefficient *γ*_tip_. This offers the advantage that the reliability of the slip-length measurement is not limited by the performance of the AGC. Extracting the coefficient of *ḣ* from eq 3, the damping coefficient can be expressed as

7 $$\gamma_{\mathrm{tip}}\left(h,b_{t},b_{s}\right)=\frac{6\pi \eta R^{2}}{h}f^{*}\left(h,b_{t},b_{s}\right)$$

Slip length *b*_s_ is then obtained by fitting eq 7 to a damping coefficient versustip–sample distance curve with *b*_s_ as the fitting parameter. We note that the slip length of the probe material, *b*_t_, needs to be precalibrated using a substrate made of the same material (*b*_t_ = *b*_s_). This method enhances slip length sensitivity by 159-fold relative to normal contact-mode AFM (see Supporting Information II for details). Consequently, this allows us to measure the slip length using a probe with a much smaller sphere.

In Figure 2(a), we show a representative damping coefficient curve as a function of tip–sample distance obtained at an interface between deionized water (Milli-Q, Millipore, USA) and highly oriented pyrolytic graphite (HOPG, SPI-1 grade; Alliance Biosystems, Japan). This measurement was performed with a colloidal probe having a 300 nm radius of curvature (Biosphere B300-NCH, Nanotools), which is about one-thirtieth of the radius typically used in past studies. SEM images of the probe are shown in Figures 2(b) and (c). The resonance frequency *f*_0_ and quality factor *Q*_bulk_ of the cantilever in water, as well as its spring constant *k*, were identified by thermal-noise analysis.^44,45^ For the measurement in Figure 2(a), we obtained *f*_0_ = 145.8 kHz, *Q*_bulk_ = 8.2, and *k* = 22 N m^–1^. The measured total damping *γ*_total_ = *γ*_tip_ + *γ*_bulk_ was consistently smaller than the theoretical curve for a no-slip assumption (*b*_s_ = 0 nm), whereas it was well fitted by the curve with *b*_s_ = 43.2 nm, demonstrating the utility of our new method.

**Figure 2:**
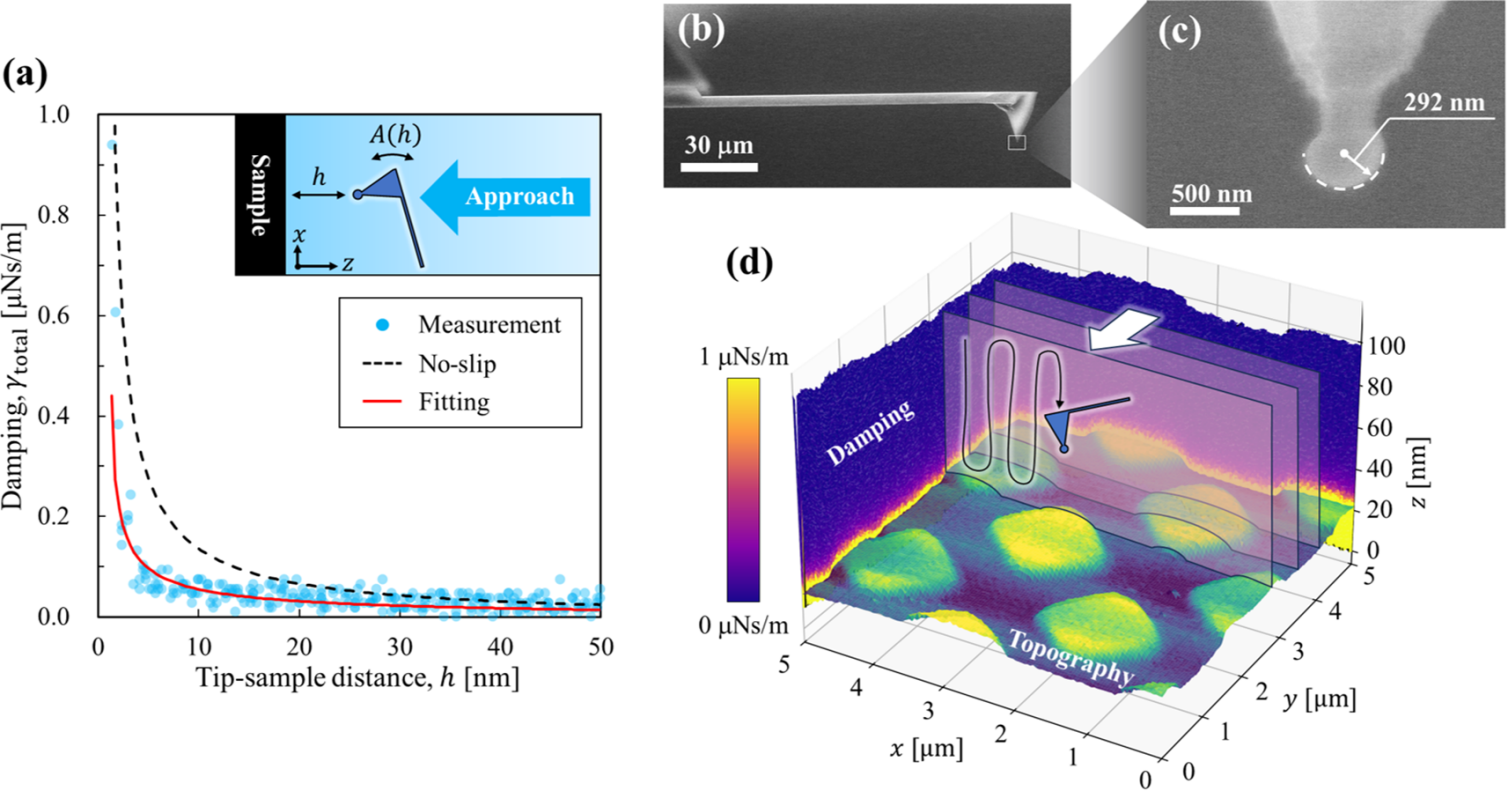
(a) Total damping coefficient *γ*_total_ as a function of probe tip–sample distance *h* measured at an HOPG-deionized water interface. Blue dots indicate the experimental data. The black dashed and red solid lines represent theoretical curves for a no-slip boundary condition (*b*_s_ = 0) and a fitted slip length (*b*_s_ = 43.2 nm), respectively. The mean absolute error for the fit was 0.014 μN s m^–1^. (b, c) SEM images of the probe with a diamond-like carbon spherical tip. (d) Schematic illustration of the simultaneous mapping of slip length and topography using FM-AFM. The color bar indicates the total damping coefficient.

Next, we extended this method to perform simultaneous nanoscale mapping of slip length and surface topography. A schematic of the measurement principle and representative three-dimensional maps of the damping coefficient are shown in Figure 2(d). Measurements were performed using the ZXY scan mode of an SPM-8100FM (Shimadzu Corp., Japan) equipped with a custom-built photothermal excitation system and closed-loop feedback scanner. At each (*x*, *y*) pixel on the sample plane, the probe was scanned in the *z*-direction while recording the cantilever deflection, amplitude, and frequency-shift. The topography was reconstructed from the deflection, whereas the slip length was determined from the damping coefficient using eq 7.

Slip length mapping was first carried out on HOPG and on a hydrophilic–hydrophobic composite substrate (oxygen-plasma-treated-silica-1H,1H,2H,2H-perfluorooctanephosphonic acid, FOPA; the fabrication procedures are in Supporting Information III-A). The contact angles of deionized-water droplets were measured immediately before the FM-AFM measurements on unpatterned reference hydrophilic and hydrophobic substrates prepared using the same process as the corresponding regions of the composite substrate, yielding 28° and 105°, respectively. The composite substrate enabled us to test artificially fabricated patterns, whereas HOPG, with its atomically flat terraces and step structures,^46^ served as a natural reference surface for verifying nanoscale spatial resolution. Immediately before measurement, the HOPG surface was cleaved with Scotch tape to expose a clean surface. Before each measurement, the cantilever was hydrophilized by atmospheric plasma treatment. In all measurements, the cantilever was controlled to oscillate with an amplitude of 2 nm. We calibrated the slip length of the probe tip by measuring a symmetric system (*b*_t_ = *b*_s_) using an atmospheric-plasma-treated diamond-like carbon (DLC) substrate, yielding a slip length of *b*_t_ = *b*_s_ = 0.0 ± 1.2 nm; a representative fitting curve is shown in Figure S1­(a).

Figure 3 clearly demonstrates that the present method enables simultaneous nanoscale visualization of topography and slip length. The topography image on HOPG in Figure 3(a) not only reveals the presence of fine terrace structures ∼ 100 nm in width, but also resolves single graphene steps with heights of 0.34 nm.^47^ On the flat area of the HOPG surface, a large slip length of 43.2 ± 5.8 nm was obtained (Figure 3(b)). Remarkably, a spherical region with significantly increased slip length compared with the surrounding area appeared, as indicated by the white triangle. As shown in Figure S2, when the tip was strongly pressed against the surface, the spherical region disappeared in the height image and the underlying HOPG substrate became visible. This suggests that the region is covered by a soft matter. Because a positive frequency shift corresponds to repulsive forces, the observed enhancement indicates that the hydrophilized probe experienced repulsion when pushing this region, a characteristic response to nanobubbles.^48,49^ The object exhibited a contact radius of 72.3 nm, a height of 22.2 nm, and a contact angle of 146°, which are in good agreement with values typically reported in previous studies.^50,51^ From these, we conclude that this feature is a nanobubble formed incidentally upon immersion. The slip length at the gas–liquid interface directly above a nanobubble was evaluated to be 345.3 ± 23.7 nm, and a representative fitting curve is shown in Figure S1­(b). This value is significantly larger than that on the surrounding HOPG surface, while remaining smaller than the slip length expected for an ideal free surface. Further characterization of slip on surface nanobubbles, including the validity of the measured value, remains an interesting topic for future studies.

**Figure 3:**
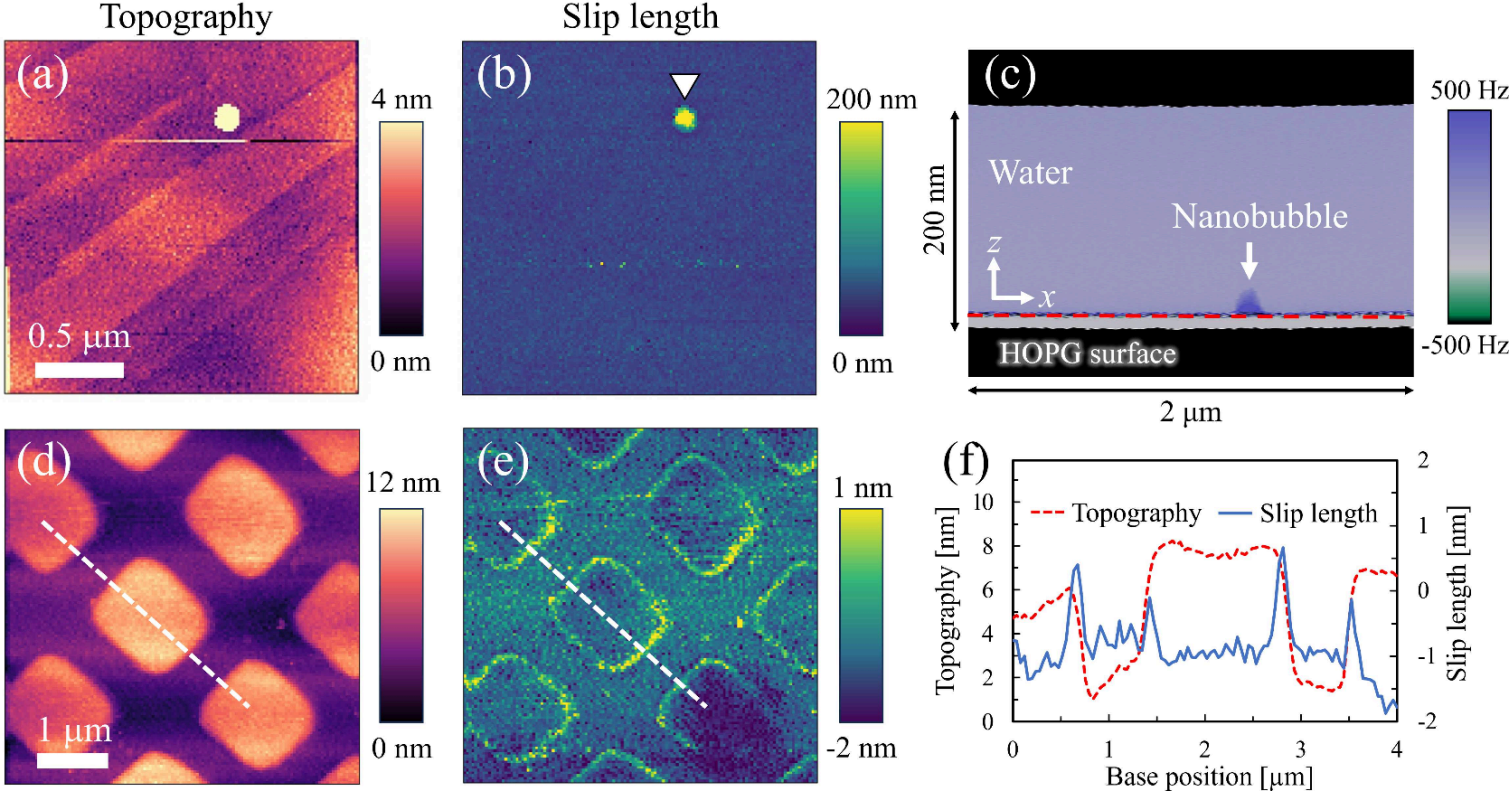
(a) Surface topography and (b) slip length maps on the HOPG surface. The average slip length was 43.2 ± 5.8 nm. (c) Z-X frequency-shift image along the location where a nanobubble was detected, as indicated by the white triangle in (b). The red dashed line corresponds to the HOPG surface. (d) Surface topography and (e) slip length maps on the FOPA-silica composite surface. The FOPA patches are 1 μm × 1 μm in lateral size and 5 nm in height. (f) Cross-sectional profiles of the topography and slip length along the white dashed lines indicated in (d) and (e).

In Figure 3(d), FOPA patches, only 5 nm in height, are clearly visualized, and their boundaries exhibit a distinct increase in slip length (Figure 3(e)) because the gap beneath the step facilitates fluid flow when the probe contacts the substrate.^52^ This increase was also confirmed in the cross-sectional profiles of the topography and slip length (Figure 3(f)), which clearly show a rise in slip length at the edges of the patch. Therefore, the larger slip lengths observed near the edges represent apparent rather than intrinsic slip. On the flat silica and FOPA regions, slip lengths of – 0.7 ± 0.6 nm and – 0.9 ± 0.6 nm, respectively, were obtained, both indicating a no-slip boundary condition, which contradicts many experimental studies reporting larger slip lengths on hydrophobic surfaces.^7,23,25^ ^,^ ^28–31^ ^,^ ^33,38,40^ A negative slip length formally means that the extrapolated zero-velocity plane lies on the liquid side of the geometrical solid–liquid interface. Such small offsets likely reflect the propagation of systematic uncertainties in the probe radius, spring constant, and distance origin into the fitted *b*_*s*_. Because the surfaces other than HOPG consistently yielded slip lengths close to zero, these apparent offsets caused by error propagation are sufficiently small compared with the slip length observed on the slippery surface. Thus, we hypothesize that the previously reported large slip lengths may have resulted from the effects of surface structures or nanobubbles, and therefore represent apparent slip lengths rather than intrinsic ones. In particular, it is well-known that surface nanobubbles are readily generated on hydrophobic surfaces upon immersion, whereas they do not form on hydrophilic surfaces.^53,54^ Therefore, on hydrophobic surfaces, the measured slip length can vary markedly depending on the presence and surface density of nanobubbles, whereas it is expected to remain close to zero regardless of the experimental conditions on hydrophilic surfaces. This is consistent with the observed trend in Figure 1.

Next, we systematically measured slip lengths on a variety of solid surfaces with contact angles ranging from 3° to 111°. The substrates used were silica, DLC, muscovite mica, HOPG, Octadecyltrichlorosilane (OTS), FOPA, 1H,1H,2H,2H-perfluorodecyltriethoxysilane (FDTS), and Teflon. DLC, OTS, FOPA, and FDTS were coated on flat Si substrates. Among the flat surfaces, Teflon exhibits one of the largest intrinsic contact angles.^55,56^ The preparation of the Teflon substrate is described in the Supporting Information III–B.

Figure 4 presents the measured slip lengths on flat substrates. The measured values and those predicted by eq 2 with *C* = 0.41 nm and *C* = 6.0 nm for all substrates investigated in this study are summarized in Table S2 of the Supporting Information. Interestingly, on all the surfaces except HOPG, the slip lengths were almost zero, in good agreement with the MD-based scaling law in eq 2 with *C* = 0.41 nm.^16^ This supports our interpretation that earlier experimental studies may have overestimated slip lengths due to the influence of nanoscale surface features. Even on the most hydrophobic sample (Teflon), the slip length was only 0.8 ± 0.8 nm, clearly indicating that the relation “hydrophobic surfaces exhibit large slip” does not hold.

**Figure 4:**
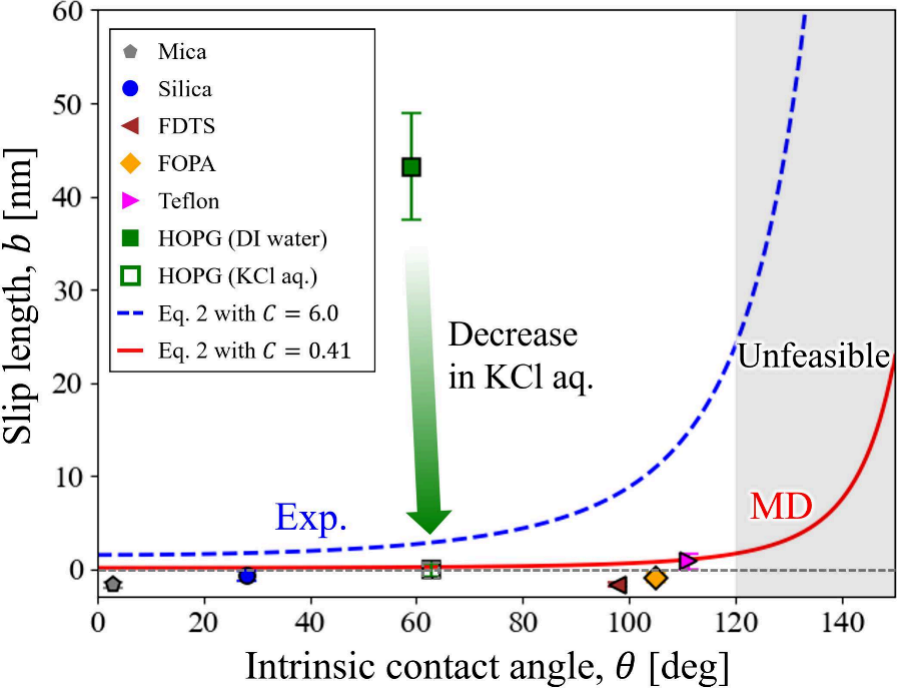
Systematic comparison of slip lengths on nanoscopically flat substrates with contact angles ranging from 3° to 111°. The error bars represent the standard deviations of the slip lengths obtained from the 128 × 128 pixels in each slip-length map. The blue and red dashed lines correspond to the theoretical scaling law of eq 2 with *C* = 6.0 nm and *C* = 0.41 nm, respectively. The gray region represents contact angles above the approximate upper range expected for chemically homogeneous flat surfaces. The corresponding topography and slip-length maps, together with the measured nanoscale roughness values, are shown in Figure S3.

In contrast, only HOPG (contact angle 59.0°) exhibited a markedly large slip length of 43.2 ± 5.8 nm, inconsistent with the scaling law. The HOPG surface was cleaved immediately before measurement and showed only moderate wettability, with a contact angle close to that reported for cleaved HOPG (64.4°).^57^ Therefore, it is unlikely to have been contaminated by hydrophobic adsorbates.^58–60^ The localized nanobubble observed in Figure 3(b) was analyzed separately and thus did not contribute to the slip length measured on the flat HOPG terrace. Therefore, we attribute the slip length on the flat HOPG terrace to the low-friction graphite-water interface. Previous MD studies have reported the uniqueness of graphite: its atomic-scale smoothness drastically reduces friction, leading to the slip length on the order of 50 nm.^61,62^ Although mica also has an atomically smooth surface, unlike graphite, which is composed solely of carbon atoms, its surface consists of four elements (K, Si, Al, and O). Such atomic-scale chemical heterogeneity leads to a more strongly corrugated interfacial energy landscape, as predicted by ab initio MD,^19^ resulting in an extremely small slip length. Therefore, our experimental results are in good agreement with past simulations.

We note that the large slip observed on planar HOPG should be distinguished from the massive slippage reported inside carbon nanotubes.^63^ As Secchi et al. demonstrated, the slip length can reach several hundred nanometers due to a curvature-enhanced effect, whereas it is much smaller on a noncurved plane, on the order of several tens of nanometers. Our results on planar HOPG are quantitatively consistent with the slip length reported by Secchi et al. for planar graphite.^63^

Interestingly, the slip length on HOPG decreased to – 0.1 ± 0.7 nm when measured in a 10 mmol L^–1^ KCl solution, indicating a no-slip boundary condition. This is likely due to interfacial charging and ion adsorption in the electrolyte solution. A recent MD study reported that even a slight charge on graphene surfaces (∼20 mV) reduces the slip length from 45 to 2 nm in an electrolyte solution.^18^ One major mechanism is that K^+^ ions selectively adsorb onto the charged graphene surface and, through their structured hydration shells, strongly pin water molecules to the surface.^64^ Because actual graphene surfaces typically have a surface potential of ∼ 20 mV,^65^ adding electrolyte is expected to significantly suppress slip. A similar reduction was observed in a 10 mmol L^–1^ NaCl solution (not shown in Figure 4, see Table S2 in Supporting Information). To our knowledge, this is the first experimental result that verifying the effect of ion adsorption on slip predicted by MD simulations. Paradoxically, these results suggest that controlling surface charge may offer a route to enhancing slip on graphite/graphene and potentially achieving appreciable slip on other surfaces, which we will investigate in future work.

In conclusion, we developed an experimental method for slip length measurement using FM-AFM and achieved a 159-fold improvement in slip length detection sensitivity. This enabled, for the first time, simultaneous nanoscale mapping of slip length and surface topography, allowing us to quantify the true slip length by excluding the influence of surface heterogeneities such as surface roughness and nanobubbles. A systematic investigation has resolved the long-standing discrepancy between experiments and MD simulations, leading to the conclusion that hydrophobic surfaces are not slippery. A large slip length in deionized water was observed only on the HOPG surface with mild wettability. This result demonstrates that slip length on flat surfaces depends more strongly on the crystalline structure than on wettability, highlighting the singular nature of graphite. The large slip length on HOPG became negligibly small when measured in electrolyte solutions, in agreement with past MD analysis. Our method substantially improves the accuracy of slip length measurements and provides, to the best of our knowledge, the only experimental technique capable of quantitatively evaluating true slip and correlating it with nanoscale surface characteristics. This breakthrough not only bridges the long-standing gap between experiments and MD simulations, but also opens a new avenue for experimentally exploring how slip length is affected by factors that are difficult to reproduce in MD, such as electric double layers, nanobubbles, and hydrogels.

## Supplementary Material



## Notes

Details of the AFM measurement principle, derivation of the relation between amplitude decay and damping, sensitivity comparison with contact-mode AFM, fabrication procedures of composite and Teflon substrates, and supplementary figures and tables (PDF)
