# Supplementary material for: Hydrophobicity Does Not Affect Water Slip: Insights from Slip Length Mapping
Source: Nano Lett. 2026 May 15;26(20):6698–704. doi: 10.1021/acs.nanolett.6c01403 (PMC13220299; doi:10.1021/acs.nanolett.6c01403)
Supplement: Supplementary file 1 [file nl6c01403_si_001.pdf]

Supporting Information
Hydrophobicity Does Not Affect Water Slip: Insights from Slip Length Mapping

Haruya Ishida,[†] Koji Takahashi,[†,‡] Vishwanath Ganesan,[¶] Nenad Miljkovic,[‡,¶,§,‖,⊥,#] and Hideaki Teshima[*,†,‡]

†*Department of Aeronautics and Astronautics, Kyushu University, Nishi-Ku, Motooka 744, Fukuoka 819-0395, Japan*

‡*International Institute for Carbon-Neutral Energy Research (WPI-I2CNER), Kyushu University, Nishi-Ku, Motooka 744, Fukuoka 819-0395, Japan*

¶*Department of Mechanical Science and Engineering, University of Illinois at Urbana-Champaign, Urbana, Illinois 61801, United States*

§*Materials Research Laboratory, University of Illinois at Urbana-Champaign, Urbana, Illinois 61801, United States*

‖*Department of Electrical and Computer Engineering, University of Illinois at Urbana-Champaign, Urbana, Illinois 61801, United States*

⊥*Institute for Sustainability, Energy and Environment (iSEE), University of Illinois at Urbana-Champaign, Urbana, Illinois 61801, United States*

#*Air Conditioning and Refrigeration Center, University of Illinois at Urbana-Champaign, Urbana, Illinois 61801, United States*

E-mail: hteshima05@aero.kyushu-u.ac.jp

I. Relation between Amplitude Decay and Damping Coefficient

The total damping coefficient, γ_{total}, acting on the probe can be expressed as the sum of the damping due to the probe sphere, γ_{tip}, and the background damping, γ_{bulk}:

$$\gamma_{\text{total}} = \gamma_{\text{tip}}(h, b_{\text{t}}, b_{\text{s}}) + \gamma_{\text{bulk}}. \tag{S1}$$

where h is probe-substrate distance, and b_{t} and b_{s} are the slip lengths at the probe and substrate surfaces, respectively. The background damping γ_{bulk} originates from various sources, such as viscous drag on the cantilever beam and friction due to thermal fluctuations.[1] However, it remains nearly constant and is therefore treated as a constant here. Using the definition of the quality factor $Q(h) = k/(\omega\gamma_{\text{total}}(h))$, Eq. S1 can be rewritten as:

$$\frac{k}{\omega Q(h)} = \gamma_{\text{tip}}(h, b_{\text{t}}, b_{\text{s}}) + \gamma_{\text{bulk}}. \tag{S2}$$

At sufficiently large $h \gg R$, the influence of the substrate becomes negligible and the hydrodynamic drag on the probe reduces to the Stokes resistance, $\gamma_{\text{tip}} = 6\pi\eta R$. Because this drag on a 300 nm spherical tip is orders of magnitude smaller than the viscous damping acting on the micrometer-scale cantilever, γ_{tip} becomes negligibly small compared to γ_{bulk}, leading to:

$$\gamma_{\text{total}} = \gamma_{\text{bulk}} = \frac{k}{\omega Q_{\text{bulk}}}. \tag{S3}$$

Combining Eqs. S2 and S3 yields:

$$\frac{Q_{\text{bulk}}}{Q(h)} = \frac{\gamma_{\text{tip}}(h, b_{\text{t}}, b_{\text{s}})}{\gamma_{\text{bulk}}} + 1. \tag{S4}$$

In normal FM-AFM, the oscillation amplitude is stabilized by automatic gain control (AGC). In the present method, AGC was disabled and the cantilever was driven at a constant power. As a result, the amplitude decay directly reflects the damping, and the following relation

between the quality factor and the amplitude holds:

$$\frac{Q_{\mathrm{bulk}}}{Q(h)} = \frac{A_{\mathrm{bulk}}}{A(h)}. \tag{S5}$$

Therefore, from Eqs. S4 and S5, the damping coefficient is related to the amplitude attenuation ratio as follows:

$$\gamma_{\mathrm{tip}}(h, b_{\mathrm{t}}, b_{\mathrm{s}}) = \gamma_{\mathrm{bulk}} \left(\frac{A_{\mathrm{bulk}}}{A(h)} - 1 \right). \tag{S6}$$

II. Comparison of Sensitivity between Conventional and Proposed Methods

We compared the sensitivity of slip length measurements between conventional contact-mode AFM and the proposed FM-AFM method. In contact-mode AFM, the cantilever deflection x is measured as a sensor voltage, and the relationship between the deflection sensor voltage V_x and x is expressed using the sensitivity s as $x = sV_x$. Furthermore, to determine the viscous drag in Eq. 3 from the restoring force of the cantilever, kx (k is the spring constant), the following relation holds:

$$F = -ksV_x = -\frac{6\pi\eta R^2 \dot{h}}{h} f^*(h, b_{\mathrm{t}}, b_{\mathrm{s}}). \tag{S7}$$

In contrast, in the FM-AFM method, the oscillation amplitude A is recorded as a sensor voltage V_A, with the relationship $A = sV_A/\sqrt{2}$. From Eqs. 6 and 7 of the manuscript, the following relation is obtained:

$$\frac{6\pi\eta R^2}{h} f^*(h, b_{\mathrm{t}}, b_{\mathrm{s}}) = \gamma_{\mathrm{bulk}} \left(\frac{V_{A,\mathrm{bulk}}}{V_A(h)} - 1 \right). \tag{S8}$$

Here, the measurement sensitivity is defined as the change in signal voltage with respect to the substrate slip length, $\partial V / \partial b_{\mathrm{s}}$. Differentiating Eqs. S7 and S8 yields the sensitivities for

each method, S_{contact} and S_{FM}:

$$S_{\text{contact}} = \frac{\partial V_x}{\partial b_{\text{s}}} = -\frac{6\pi\eta R^2 \dot{h}}{ksh}\frac{\partial f^*}{\partial b_{\text{s}}}. \tag{S9}$$

$$S_{\text{FM}} = \frac{\partial V_A}{\partial b_{\text{s}}} = -\frac{6\pi\eta R^2 V_{A,\text{bulk}}}{h\gamma_{\text{bulk}}}\left(1 + \frac{6\pi\eta R^2 f^*}{h\gamma_{\text{bulk}}}\right)^{-2}\frac{\partial f^*}{\partial b_{\text{s}}}. \tag{S10}$$

From Eqs. S9 and S10, the ratio of sensitivities is given by:

$$\frac{S_{\text{FM}}}{S_{\text{contact}}} = \frac{V_{A,\text{bulk}} ks}{\gamma_{\text{bulk}} \dot{h}}\left(1 + \frac{6\pi\eta R^2 f^*}{h\gamma_{\text{bulk}}}\right)^{-2}. \tag{S11}$$

The sensitivity ratio was calculated to be $S_{\text{FM}}/S_{\text{contact}} = 159$ using typical physical parameters in the measurements: $V_{A,\text{bulk}} = 0.2\,\text{V}$, $k = 20\,\text{N}\,\text{m}^{-1}$, $s = 10\,\text{nm}\,\text{V}^{-1}$, $\gamma_{\text{bulk}} = 2.3\,\mu\text{N}\,\text{s}\,\text{m}^{-1}$, $\dot{h} = 100\,\mu\text{m}\,\text{s}^{-1}$, $R = 300\,\text{nm}$, $\eta = 1\,\text{mPa}\,\text{s}$, $f^* = 0.63$, and $h = 10\,\text{nm}$. The f^* value corresponds to the slip length of $b_{\text{t}} = 0\,\text{nm}$ and $b_{\text{s}} = 10\,\text{nm}$, and the tip substrate distance $h = 10\,\text{nm}$ is chosen as a representative value among the range of distances used in the calculation. This indicates that the present method is 159 times more sensitive to slip length than the conventional approach. Consequently, slip length can be measured using much smaller colloidal probes than before.

III. Materials

A. Fabrication of Hydrophilic/Hydrophobic Composite Substrates

A thermally grown silica surface on a Si wafer was spin-coated with an electron-beam resist (ZEP520A, ZEON Corporation, Japan) at $5000\,\text{min}^{-1}$ to form a 300 nm-thick film. Using an electron-beam lithography system (JSM-6360, JEOL Ltd., Japan / BEAM DRAW, Tokyo Technology Inc., Japan), square patterns of $1\,\mu\text{m}\times 1\,\mu\text{m}$ were patterned with a pitch of $2\,\mu\text{m}$, and the resist in the exposed areas was removed during development. Subsequently, a 5 nm-thick indium tin oxide (ITO) film was deposited by magnetron sputtering (MS-4B-3T, Cosmo System Co., Ltd., Japan). The electron-beam resist was then lifted off by rinsing

in dimethylacetamide for 5 min, leaving ITO patches on the silica substrate. The patterned ITO substrate was treated with O_2 plasma (PR500, Yamato Scientific Co., Japan) and then immersed in a $1\,\mathrm{mmol\,L^{-1}}$ ethanol solution of 1H,1H,2H,2H-perfluorooctanephosphonic acid (FOPA) for 1 h to form a self-assembled monolayer (SAM). Since FOPA SAMs are easily hydrolyzed on silica but remain stable on ITO surfaces and maintain hydrophobicity in water for several hours,[2,3] the fabricated composite substrate was immersed in water for 3 h to selectively remove FOPA from the silica surface. Contact angles of the hydrophilic and hydrophobic regions were evaluated immediately before the FM-AFM measurements using unpatterned reference substrates that underwent the corresponding O_2-plasma treatment, FOPA/ethanol-solution immersion, and subsequent water-immersion steps used to prepare the composite substrate.

B. Fabrication of Teflon Substrates

Silica substrates cut into $1\,\mathrm{cm}\times 1\,\mathrm{cm}$ were ultrasonically cleaned in acetone (US-1KS, SND Co., Ltd., Japan) and treated with O_2 plasma. After baking at 120 °C for 5 min to remove surface moisture, the substrates were immersed for 1 h in a 0.05 % (w/w) toluene solution of FDTS to form an FDTS monolayer. On top of the FDTS-treated substrate, a 0.6 % (w/w) solution of Teflon AF1600X (DuPont Inc., Wilmington, DE, USA) in FC-770 (3M, St. Paul, MN, USA) was dropped, spin-coated at $4000\,\mathrm{min^{-1}}$ for 1 min, and baked at 175 °C for 10 min. The FDTS underlayer was used to improve the adhesion of the Teflon coating.[4]

Table S1: Literature values of contact angle and slip length for various solid-liquid interfaces

Author	Method	Substrate	Contact Angle θ (°)	Slip length (nm)
Bonaccurso et al. 2002[5]	AFM	Mica	0	8.5 ± 0.5
Vinogradova & Yakubov 2003[6]	AFM	Silica	0	0 ± 1
		Polystyrene	86–92	4 ± 1
Cho et al. 2004[7]	AFM	OTS	97.5	30
Cottin-Bizonne et al. 2005[8]	AFM	OTS-Pyrex	105	19 ± 2
Maali et al. 2008[9]	AFM	HOPG	74	8 ± 2
		Mica	0	2
Bouzigues et al. 2008[10]	TIRF-PIV	Silica	20	0 ± 10
		OTS	95	38 ± 6
		Silica	20	3 ± 7
		OTS	95	29 ± 11
Cottin-Bizonne et al. 2008[11]	SFA	OTS-Pyrex	105	17 ± 2
		Pyrex	3	0
		DPPC monolayer	95	10 ± 2
Lasne et al. 2008[12]	TIRF-PIV	OTS	90	45 ± 15
		Silica	0	0
Bhushan et al. 2009[13]	AFM	Mica	0	0
		n-hexatriacontane	91 ± 2	43 ± 10
		Lotus wax	167 ± 0.7	236 ± 18
Zhu et al. 2011[14]	AFM	OTS	112	26 ± 3
Jing & Bhushan 2013[15]	AFM	OTS	106 ± 2	28 ± 5
Ortiz-Young et al. 2013[16]	AFM	Mica	4 ± 3	0
		GO	48 ± 3	0.24 ± 0.38
		DLC	66 ± 6	0.55 ± 1.37
		Si	76 ± 4	1 ± 1.7
		HOPG	85 ± 4	12 ± 3.3
Xue et al. 2015[17]	QCM	DLC	75–100	2.4
		MUOH	40–80	1.9
		MUOH	0–60	1.2
		MUOH	0–50	0.7
Ahmad et al. 2015[18]	AFM	Si	0	0
		OTS	108 ± 3	0
Radha et al. 2016[19]	Channel	Graphene	55–85	60
Zhang et al. 2021[20]	AFM	Mica	10	0
		Talc	75	95
Keerthi et al. 2021[21]	Channel	Graphene	62	60
		hBN	62	1
Li et al. 2022[22]	AFM	OTS	100	13.4 ± 3
		SiO_2	24.9	0.9 ± 1.2
		HOPG	75 ± 5	4.3 ± 3.5
Chen et al. 2022[23]	Channel	Graphene	83.7	40 ± 4
		N/A	83.7	33 ± 3
Han et al. 2025[24]	AFM	SiO_2	41.2 ± 0.7	4.3
		APTES	68.2 ± 1.9	9.4
		OTS	108.5 ± 0.1	29.1
		FDTES	125.7 ± 1.8	65.3

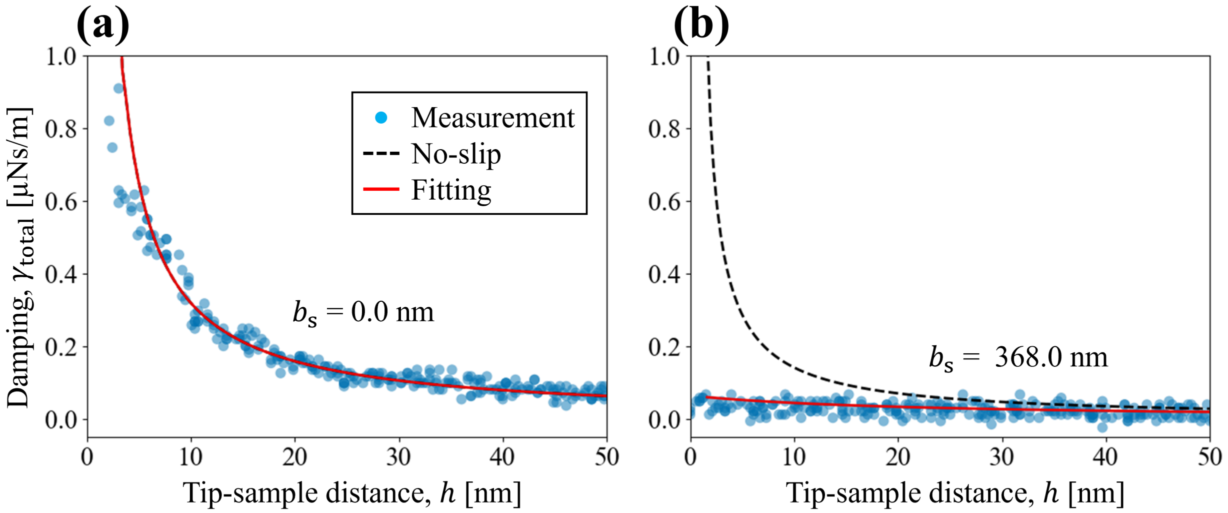

Figure S1: Representative damping-coefficient fitting curves for (a) an atmospheric-plasma-treated DLC substrate and (b) a location above a nanobubble. The slip lengths shown in the panels were obtained from these representative single-point fits and therefore do not necessarily coincide with the mean values reported in the main text: 0.0 ± 1.2 nm for DLC and 345.3 ± 23.7 nm for nanobubbles. The mean absolute errors were $0.016\,\mu\mathrm{N\,s\,m^{-1}}$ and $0.014\,\mu\mathrm{N\,s\,m^{-1}}$ for (a) and (b), respectively.

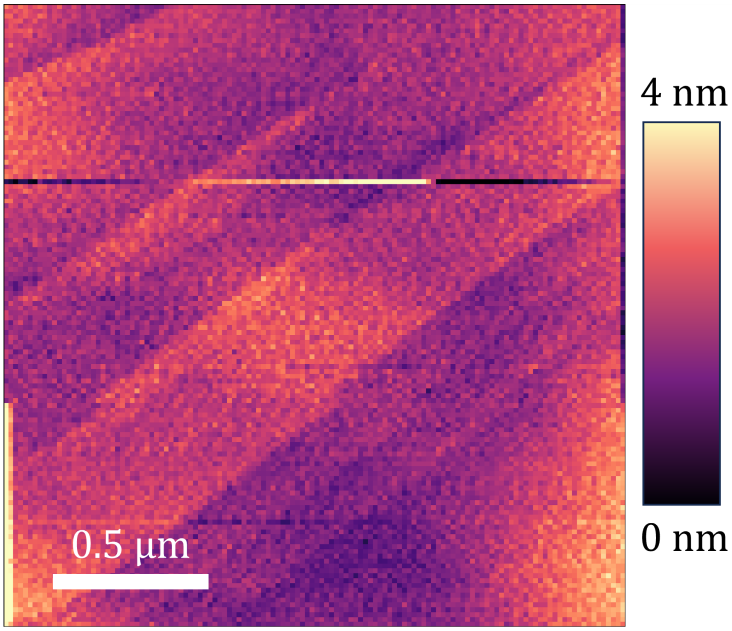

Figure S2: Height image acquired while the tip was strongly pressed against the surface, showing disappearance of the bubble-like feature and exposure of the underlying HOPG substrate.

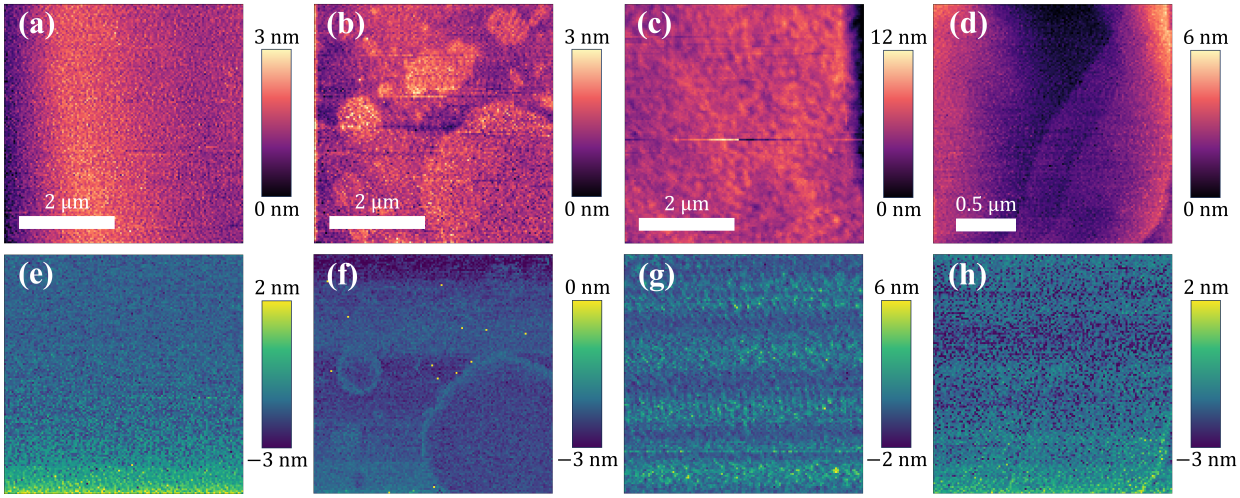

Figure S3: (a–d) Topography and (e–h) corresponding slip-length maps of (a, e) mica, (b, f) FDTS, (c, g) Teflon, and (d, h) HOPG. The R_a values were 0.27 nm, 0.29 nm, 0.64 nm, and 0.35 nm for each surface, respectively. Only the result for HOPG (d, h) was obtained in a 10 mmol L^{-1} KCl solution and the others were in DI water.

Table S2: Measured slip lengths and values predicted by Eq. 2 for the substrates investigated in this study.

Substrate	Contact Angle (°)	b predicted by $C = 0.41$ nm (nm)	b predicted by $C = 6.0$ nm (nm)	Measured b (nm)
Mica	< 3	~ 0.10	~ 1.50	-1.7 ± 0.3
Oxygen plasma-treated silica	28.1	0.12	1.69	-0.7 ± 0.6
FDTS	97.9	0.55	8.06	-1.7 ± 0.2
FOPA	105.0	0.75	10.92	-0.9 ± 0.6
Teflon	111.1	1.00	14.65	0.8 ± 0.8
HOPG (DI water)	59.0	0.18	2.61	43.2 ± 5.8
HOPG (10 mM KCl aq.)	62.8	0.19	2.83	-0.1 ± 0.7
HOPG (10 mM NaCl aq.)	57.0	0.17	2.51	-2.3 ± 0.6

Supporting References

(1) Gauthier, M.; Tsukada, M. Theory of noncontact dissipation force microscopy. *Phys. Rev. B Condens. Matter* **1999**, *60*, 11716–11722.

(2) Thissen, P.; Seitz, O.; Chabal, Y. J. Wet chemical surface functionalization of oxide-free silicon. *Prog. Surf. Sci.* **2012**, *87*, 272–290.

(3) Jo, K.; Yu, H.-Z.; Yang, H. Formation kinetics and stability of phosphonate self-assembled monolayers on indium–tin oxide. *Electrochim. Acta* **2011**, *56*, 4828–4833.

(4) Makohliso, S. A.; Giovangrandi, L.; Léonard, D.; Mathieu, H. J.; Ilegems, M.; Aebischer, P. Application of Teflon-AF® thin films for bio-patterning of neural cell adhesion1S.A. Makholiso and L. Giovangrandi contributed equally to this work.1. *Biosens. Bioelectron.* **1998**, *13*, 1227–1235.

(5) Bonaccurso, E.; Kappl, M.; Butt, H.-J. Hydrodynamic force measurements: boundary slip of water on hydrophilic surfaces and electrokinetic effects. *Phys. Rev. Lett.* **2002**, *88*, 076103.

(6) Vinogradova, O. I.; Yakubov, G. E. Dynamic Effects on Force Measurements. 2. Lubrication and the Atomic Force Microscope. *Langmuir* **2003**, *19*, 1227–1234.

(7) Cho, J.-H. J.; Law, B. M.; Rieutord, F. Dipole-dependent slip of Newtonian liquids at smooth solid hydrophobic surfaces. *Phys. Rev. Lett.* **2004**, *92*, 166102.

(8) Cottin-Bizonne, C.; Cross, B.; Steinberger, A.; Charlaix, E. Boundary slip on smooth hydrophobic surfaces: intrinsic effects and possible artifacts. *Phys. Rev. Lett.* **2005**, *94*, 056102.

(9) Maali, A.; Cohen-Bouhacina, T.; Kellay, H. Measurement of the slip length of water flow on graphite surface. *Appl. Phys. Lett.* **2008**, *92*, 053101.

(10) Bouzigues, C. I.; Tabeling, P.; Bocquet, L. Nanofluidics in the Debye layer at hydrophilic and hydrophobic surfaces. *Phys. Rev. Lett.* **2008**, *101*, 114503.

(11) Cottin-Bizonne, C.; Steinberger, A.; Cross, B.; Raccurt, O.; Charlaix, E. Nanohydrodynamics: the intrinsic flow boundary condition on smooth surfaces. *Langmuir* **2008**, *24*, 1165–1172.

(12) Lasne, D.; Maali, A.; Amarouchene, Y.; Cognet, L.; Lounis, B.; Kellay, H. Velocity profiles of water flowing past solid glass surfaces using fluorescent nanoparticles and molecules as velocity probes. *Phys. Rev. Lett.* **2008**, *100*, 214502.

(13) Bhushan, B.; Wang, Y.; Maali, A. Boundary slip study on hydrophilic, hydrophobic, and superhydrophobic surfaces with dynamic atomic force microscopy. *Langmuir* **2009**, *25*, 8117–8121.

(14) Zhu, L.; Attard, P.; Neto, C. Reliable measurements of interfacial slip by colloid probe atomic force microscopy. II. Hydrodynamic force measurements. *Langmuir* **2011**, *27*, 6712–6719.

(15) Jing, D.; Bhushan, B. Quantification of surface charge density and its effect on boundary slip. *Langmuir* **2013**, *29*, 6953–6963.

(16) Ortiz-Young, D.; Chiu, H.-C.; Kim, S.; Voïtchovsky, K.; Riedo, E. The interplay between apparent viscosity and wettability in nanoconfined water. *Nat. Commun.* **2013**, *4*, 2482.

(17) Xue, Y.; Wu, Y.; Pei, X.; Duan, H.; Xue, Q.; Zhou, F. How solid-liquid adhesive property regulates liquid slippage on solid surfaces? *Langmuir* **2015**, *31*, 226–232.

(18) Ahmad, K.; Zhao, X.; Pan, Y.; Wang, W.; Huang, Y. Atomic force microscopy measurement of slip on smooth hydrophobic surfaces and possible artifacts. *J. Phys. Chem. C Nanomater. Interfaces* **2015**, *119*, 12531–12537.

(19) Radha, B. et al. Molecular transport through capillaries made with atomic-scale precision. *Nature* **2016**, *538*, 222–225.

(20) Zhang, C.; Wang, X.; Jin, J.; Li, L.; Miller, J. D. AFM Slip Length Measurements for Water at Selected Phyllosilicate Surfaces. *Colloids and Interfaces* **2021**, *5*, 44.

(21) Keerthi, A.; Goutham, S.; You, Y.; Iamprasertkun, P.; Dryfe, R. A. W.; Geim, A. K.; Radha, B. Water friction in nanofluidic channels made from two-dimensional crystals. *Nat. Commun.* **2021**, *12*, 3092.

(22) Li, H.; Xu, Z.; Ma, C.; Ma, M. Translucency and negative temperature-dependence for the slip length of water on graphene. *Nanoscale* **2022**, *14*, 14636–14644.

(23) Chen, K.-T.; Li, Q.-Y.; Omori, T.; Yamaguchi, Y.; Ikuta, T.; Takahashi, K. Slip length measurement in rectangular graphene nanochannels with a 3D flow analysis. *Carbon N. Y.* **2022**, *189*, 162–172.

(24) Han, Y.; Zhang, R.; Wu, Z.; Liu, H.; Luo, J.; Liu, D. Electronic-mediated slip regulation at MoS2-water interface via self-assembled monolayers. *ACS Nano* **2025**, *19*, 38792–38802.
